# Acute kidney injury among medical and surgical in-patients in the Cape Coast Teaching Hospital, Cape Coast, Ghana: a prospective cross-sectional study

**DOI:** 10.4314/ahs.v21i2.40

**Published:** 2021-06

**Authors:** Richard KD Ephraim, Yaw A Awuku, Ignatious Tetteh-Ameh, Charles Baffe, Godsway Aglagoh, Victor A Ogunajo, Kizito Owusu-Ansah, Prince Adoba, Samuel Kumordzi, Joshua Quarshie

**Affiliations:** 1 Department of Medical Laboratory Science, School of Allied Health Sciences, College of Health and Allied Sciences, University of Cape Coast, Ghana; 2 Department of Medicine and Therapeutics, School of Medical Sciences, College of Health and Allied Sciences, University of Cape Coast, Ghana; 3 Department of Molecular Medicine, School of Medical Sciences, College of Health Sciences, Kwame Nkrumah University of Science and Technology, Ghana

**Keywords:** Acute kidney injury, KDIGO, medical, surgical, hypertension, liver disease

## Abstract

**Background:**

Acute kidney injury (AKI) is a syndrome associated with high morbidity, mortality and high hospital costs. Despite its adverse clinical and economic effects, only a few studies have reported reliable estimates on the incidence of AKI in sub-Sahara Africa. We assessed the incidence and associated factors of AKI among medical and surgical patients admitted to a tertiary hospital in Ghana.

**Methods:**

A prospective cross-sectional study was conducted among one hundred and forty-five (145) consecutive patients admitted to the medical and the surgical wards at the Cape Coast Teaching Hospital (CCTH), Cape Coast, Ghana from April 2017 to April 2018. Socio-demographic and clinical information were collected using structured questionnaires. AKI was diagnosed and staged with the KDIGO guideline, using admission serum creatinine as baseline kidney function.

**Results:**

The mean age of the study participants was 46.6±17.7 years, whilst the male:female ratio was 68:77. The overall incidence of AKI among the participants was 15.9% (95% CI: 10.33 – 22.84%). Stage 1 AKI occurred in 56.5% of the participants, whilst stages 2 and 3 AKI respectively occurred among 4.1% and 2.8% of respondents. About 20% of the participants in the medical ward developed AKI (n= 15) whilst 12% of those in surgical ward developed AKI (n= 8). Among the participants admitted to the medical ward, 60.0%, 26.7% and 13.3% had stages 1, 2 and 3 AKI respectively. Whilst 50.0%, 25.0% and 25.0% respectively developed stages 1, 2 and 3 AKI in the surgical ward. Medical patients with AKI had hypertension (40%), followed by liver disease (33.3%); 37.5% of surgical inpatients had gastrointestinal (GI) disorders.

**Conclusion:**

The incidence of AKI is high among medical and surgical patients in-patients in the CCTH, Ghana, with hypertension and liver disease as major comorbidities.

## Introduction

Acute kidney injury (AKI) is a syndrome defined by a sudden decrease in glomerular function^1^. It is a common clinical condition in all countries of the world, irrespective of economic status. The syndrome is associated with high morbidity, mortality and high hospital costs 1. Furthermore, an episode of AKI can progress to chronic kidney disease (CKD) or end- stage kidney disease (ESKD) 2, 3.

The global burden of AKI has been estimated to be 13.3 million cases per year, 11.3 million of which are in low- to middle-income countries (LMICs), and responsible for up to 1.4 million deaths per year. Furthermore, AKI-related complications account for up to 3% of hospital admissions in general health care facilities in low resource settings 4. A study by Hoste et al. 5 also reported a 1.7% incidence among hospitalized patients in West Africa.

Community-acquired AKI in developing countries is mostly common in rural areas, and its true prevalence and leading causes are not well known, owing to underreporting, limited diagnostic capacity, and lack of awareness by health care workers 4, 6, 7.

In contrast, the cause of hospital-acquired AKI in LMICs, which has been described primarily in large urban centers, is similar to the causes in more affluent countries. It includes post-surgical complications, hemorrhage, infections, septic shock, and drug toxicity. Although peri-operative AKI can be prevented through early identification of risk factors and prompt institution of appropriate therapy during the peri-operative period, evidence on the incidence of AKI and its risk factors among patients undergoing surgery in the sub-Saharan African region is still evolving 8,9. AKI is also on the rise in LMICs resource countries such as Ghana due to the increasing prevalence of hypertension and widespread usage of poorly prepared traditional/herbal preparations, which are highly associated with AKI 10.

The costs of renal-replacement therapies are prohibitively high in most LMICs, hence prevention and early detection of kidney injury are often the realistic way to decrease its severe impact on morbidity and mortality 11. However, further studies are needed to evaluate the current burden and studies of AKI, to guide public health interventions and improvement in clinical governance. This study therefore used the Kidney Disease Improving Global Outcomes (KDIGO) guideline to examine the incidence, and associated socio demographic and clinical factors of AKI among medical and surgical patients admitted to a tertiary hospital in Ghana.

## Methods

### Study setting and Design

This was a prospective cross-sectional single-centre study that was conducted from April 2017 to April 2018 among medical and surgical in-patients, excluding the intensive care unit at the Cape Coast Teaching Hospital (CCTH). The hospital is located in Cape Coast in the central region of Ghana. The 400-bed hospital serves as the main referral facility in that part of the country and as a centre for teaching and learning for several medical, nursing and allied health students.

### Study Population/ Sample size

A total of 145 consenting consecutive participants comprising of 76 and 69 patients admitted to the medical and the surgical wards respectively were recruited for the study.

Cochran's sample size formula was used to determine the total participants to be enrolled. Using an acute kidney (AKI) prevalence of 10.5% reported by Cruz et al.^12^ with a 5% margin of error and a 95% confidence interval, a sample size of 150 was obtained. The sample size was calculated as shown below;

N=Z^2^p(1-p)/d^2^

Where;

N represents the estimated sample size

Z represents the constant for 95% confidence interval given as 1.96

p represents the average prevalence of AKI of 11% obtained from a study conducted by Medve et al. (2011)^13^. d represents the percentage margin of error taken as 5%.

By the calculation above, the actual sample size was supposed to have been 150 recruits, however, 2 died and 3 were lost to follow-up after baseline sampling.

### Eligibility criteria

All newly admitted medical and surgical patients above 18 years who gave informed consent were included in the study.

### Exclusion criteria

Patients on dialysis, patients with CKD (hypertensive nephropathy or diabetic nephropathy) and those with obstructive uropathy were excluded.

### Ethical considerations

The study was undertaken after approval by the institutional review board at the University of Cape Coast through the Institutional review board (IRB) of Medical Laboratory Science, and also by the Cape Coast Teaching Hospital Ethical Review Committee. The protocol identification number of the ethical clearance for the study was CCTHERC/RS/EC/2017/13. Written informed consent of each participant was obtained prior to his/her inclusion to partake in the study and had the freedom to withdraw from the study at any given time. Participants were assured of strict confidentiality.

### Measurement of blood pressure

Blood pressure of the participants were measured by trained personnel using a mercury sphygmomanometer (ACCOSON) after patients have rested for 5 mins. The mean values of duplicate measurements were recorded.

### Patient recruitment and data collection

After being availed of the information about the study, the demographic data (age and gender), past clinical illness, cause of admission, blood pressure and type of medication was recorded using a structured questionnaire that was administered to each consenting participant.

### Measurement of blood pressure

Blood pressure of the participants was measured by trained personnel using a mercury sphygmomanometer (ACCOSON) after patients have rested for 5 mins. The mean values of duplicate measurements were recorded. ...and were assured of strict confidentiality.

### Measurement of blood pressure, sample collection and laboratory procedures

Blood pressure of the participants were measured by trained personnel using a mercury sphygmomanometer (ACCOSON, England) after patients have rested for 5 mins. The mean values of duplicate measurements was recorded.

About 3ml of venous blood was taken twice from each participant, one on admission (baseline) and then 48 hours of admission, into gel separator tubes, allowed to clot and on centrifuged for 10 minutes. The serum was separated and stored in a freezer at -20 degrees centigrade until analysis.

### Estimation of serum urea and creatinine

Serum urea and creatinine were assayed with a chemistry analyser (Shenzhen Mindray BS-120, Shenzhen Mindray Bio-Medical Electronics Co., Ltd. China). The principles for the analysis of urea and creatinine were based on the kinetic method 14, and the Jaffe's method^15^ respectively.

### Definition and Staging of AKI

The first sCr value upon hospitalization was defined as baseline sCr. An increase in serum creatinine level ≥0.3 mg/dL (26.5 µmol/L) within 48hr of participants admission indicate the presence of AKI 16,17,18.

AKI among the participants was defined and staged using the Kidney Disease Improving Global Outcomes guideline^19^. The admission serum creatinine value was defined using baseline creatinine since previous values for most participants were absent.

### Data analysis

Data collected was entered into Microsoft Excel spread sheet 2016, validated and analysed using Statistical Package for Social Sciences version 24.0 (SPSS 24.0) (IBM, USA). The incidence and AKI stages were illustrated in bar graphs. Categorical variables were expressed in numbers and percentages. Continuous variables such as age were reported using mean and standard deviation. Chi square test was used to compare categorical variables and independent t-test used to compare means between groups. For all comparisons, P-value <0.05 was considered statistically significant.

## Results

Table 1 shows the general characteristics of study participants. The mean age of the participants was 46.6±17.7 years, with majority (24.8%) within 41 to 50 years. Most of the medical patients were females (n= 45 / 59.2%) and 37 / 53.6% of the surgical patients were males. A greater proportion were married (59.3%) and had informal education (62.8%). Hypertension was present in 15.2% of the participants (17.1% medical and 13% surgical). About 3% and 20% had history of smoking and alcohol intake respectively. No significant difference was found in the mean systolic blood pressure (SBP), diastolic blood pressure (DBP), baseline creatinine and urea between the medical and surgical patients (P>0.05).

**Table 1:** General characteristics of study participants

	Total	Medical	Surgical	P-value
Variables	(N=145)	(N=76)	(N=69)	
***Age (years)***	46.6±17.7	46.6±18.9	46.6±16.5	0.996
***Age group (years)***				
≤20	9 (6.2)	7 (9.2)	2 (2.9)	0.340
21–30	24 (16.6)	13 (17.1)	11 (15.9)	
31–40	20 (13.8)	8 (10.5)	12 (17.4)	
41–50	36 (24.8)	18 (23.7)	18 (26.1)	
51–60	22 (15.2)	9 (11.8)	13 (18.8)	
61–70	19 (13.1)	13 (17.1)	6 (8.7)	
>70	15 (10.3)	8 (10.5)	7 (10.1)	
***Sex***				0.122
Male	68 (46.9)	31 (40.8)	37 (53.6)	
Female	77 (53.1)	45 (59.2)	32 (46.4)	
***MS***				0.248
Single	37 (25.5)	17 (22.4)	20 (29.0)	
Married	86 (59.3)	47 (61.8)	39 (56.5)	
Divorced	13 (9.0)	5 (6.6)	8 (11.6)	
Widowed	9 (6.2)	7 (9.2)	2 (2.9)	
***Occupation***				0.252
Unemployed	23 (15.9)	14 (18.4)	9 (13.0)	
Informal	91 (62.8)	43 (56.6)	48 (69.6)	
Formal	19 (13.1)	10 (13.2)	9 (13.0)	
Student	12 (8.3)	9 (11.8)	3 (4.3)	
***BP***				0.100
Optimal	43 (29.7)	29 (38.2)	14 (20.3)	
Normal	37 (25.5)	15 (19.7)	22 (31.9)	
Prehypertension	30 (20.7)	14 (18.4)	16 (23.2)	
Hypertension	22 (15.2)	13 (17.1)	9 (13.0)	
ISH	13 (9.0)	5 (6.6)	8 (11.6)	
***Smoking***				0.730
No	140 (96.6)	73 (96.1)	67 (97.1)	
Yes	5 (3.4)	3 (3.9)	2 (2.9)	
***Alcohol intake***				0.618
No	116 (80.0)	62 (81.6)	54 (78.3)	
Yes	29 (20.0)	14 (18.4)	15 (21.7)	
***SBP (mmHg)***	126.81±20.52	126.30±24.17	127.38±15.70	0.754
***DBP (mmHg)***	79.43±13.71	79.22±16.02	79.67±10.72	0.847
***Baseline Creatinine*** ***(µmol/l)***	129.35±205.79	158.08±250.24	97.69±136.64	0.078
***Baseline Urea (mmol/l)***	5.50±4.56	5.23±4.64	5.78±4.48	0.469

MS: Marital status; BP: Blood Pressure, ISH: Isolated Systolic Hypertension

**Figure 1:**
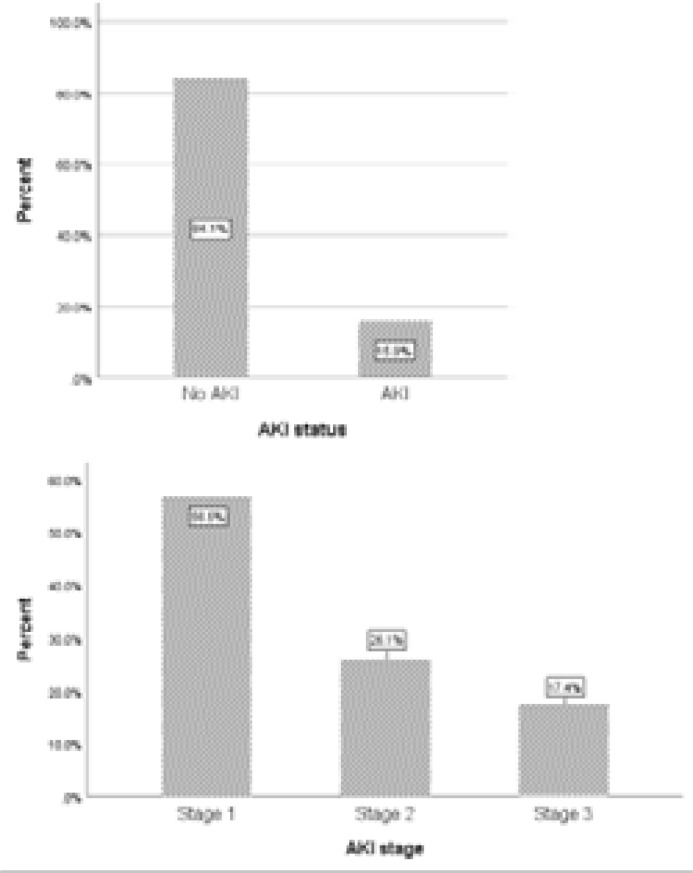
Incidence of AKI and stages among study participants

Of the 145 participants, 23 had AKI based on the KDIGO guideline giving an overall incidence of AKI among the participants to be 15.9% (95% CI: 10.33 – 22.84%), Figure 1A . From Figure 1B, stage 1 AKI was found in 56.5% (13/23) of the participants, with stage 2 and stage 3 AKI also present in 26.1% (6/23) and 17.4% (4/23) respectively (Figure 1B).

**Figure 2:**
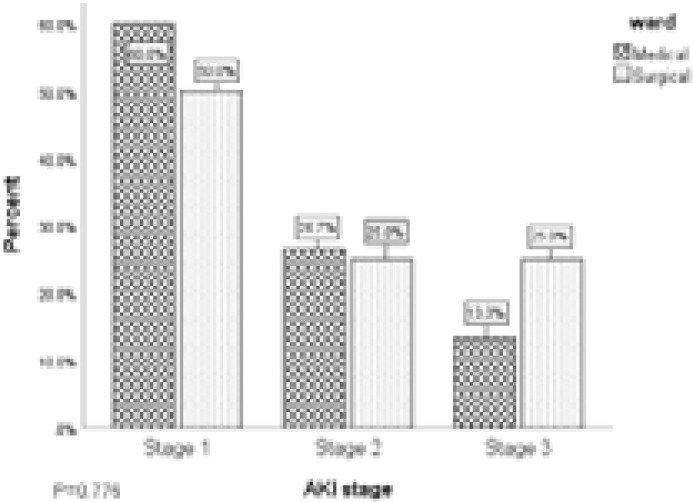
AKI stages among study participants in relation to type of ward

AKI stages among study participants in relation to type of ward is illustrated in Figure 2. Among the participants admitted to the medical ward, 60.0% (9/15), 26.7% (4/15) and 13.3% (2/15) had stages 1, 2 and 3 AKI respectively. Also, of the 69 surgical patients, 50.0% (4/8), 25.0% (2/8) and 25.0% (2/8) developed stages 1, 2 and 3 AKI respectively. No significant difference was found in the proportion of participants with AKI in relation to type of ward (P=0.521).

Table 2 describes the socio-demographic, clinical and biochemical characteristics of study participants in relation to AKI status. The mean age of participants who developed AKI was comparable to those without AKI (P=0.729). Majority of participants who developed AKI were within 41–50 years (39.1%) and females (52.2%). Also, 26.1% and 87% of those who developed AKI had hypertension and history of smoking respectively. No significant difference was found in mean SBP, DBP and baseline urea between participants who developed AKI and those without AKI (P>0.05).

**Table 2:** Socio-demographic, clinical and biochemical characteristics of study participants in relation to AKI status

Variables	No AKI (N=122)	AKI (N=23)	P-value
**Age (years)**	46.8±18.3	45.4±14.9	0.729
**Age group (years)**			0.434
≤20	8 (6.6)	1 (4.3)	
21–30	22 (18.0)	2 (8.7)	
31–40	15 (12.3)	5 (21.7)	
41–50	27 (22.1)	9 (39.1)	
51–60	20 (16.4)	2 (8.7)	
61–70	17 (13.9)	2 (8.7)	
>70	13 (10.7)	2 (8.7)	
Sex			0.922
Male	57 (46.7)	11 (47.8)	
Female	65 (53.3)	12 (52.2)	
MS			0.683
Single	33 (27.0)	4 (17.4)	
Married	72 (59.0)	14 (60.9)	
Divorced	10 (8.2)	3 (13.0)	
Widowed	7 (5.7)	2 (8.7)	
Occupation			0.838
Unemployed	20 (16.4)	3 (13.0)	
Informal	75 (61.5)	16 (69.6)	
Formal	16 (13.1)	3 (13.0)	
Student	11 (9.0)	1 (4.3)	
BP			0.411
Optimal	35 (28.7)	8 (34.8)	
Normal	32 (26.2)	5 (21.7)	
Prehypertension	27 (22.1)	3 (13.0)	
Hypertension	16 (13.1)	6 (26.1)	
ISH	12 (9.8)	1 (4.3)	
Smoking			**0.006**
No	120 (98.4)	20 (87.0)	
Yes	2 (1.6)	3 (13.0)	
Alcohol intake			0.426
No	99 (81.1)	17 (73.9)	
Yes	23 (18.9)	6 (26.1)	
***SBP (mmHg)***	126.97±19.14	126.00±27.22	0.837
***DBP (mmHg)***	79.61±13.50	78.52±15.06	0.729
***Baseline Urea (mmol/l)***	5.26±4.54	6.73±4.57	0.158

The mean ages of the surgical patients and medical patients who developed AKI were similar (P=0.757). Most of the medical patients who developed AKI were females (9/15, 60.0%) while most of the surgical patients with AKI were males (5/8, 62.5%). Majority of both surgical and medical patients with AKI had informal occupation. Hypertension was present in 33.3% of the medical patients with AKI and 12.5% of the surgical patients with AKI. Among the medical patients with AKI, two-fifth were diagnosed of hypertension (40%), followed by liver disease (33.3%), unknown condition (13.3%) and 6.7% had haemoglobinopathy and lung disease each. Of the 8 surgical patients with AKI, majority (50%) had unknown condition, 3 (37.5%) had GI disorder and 1(12.5%) had trauma.

Table 4 examines AKI, type of ward, and the association between the type of AKI, type of ward, socio-demographic and clinical characteristics of the studied participants. Smoking showed a significant association with AKI with 13% of AKI patients on the medical ward being smokers.

**Table 4:** Association between AKI, type of ward and socio-demographic and clinical characteristics of studied participants

	Medical			Surgical		
	AKI	Non-AKI	P-value	AKI	Non AKI	P-value
≤20	1 (6.7)	6 (9.8)	0.750	0 (0.0)	2 (3.3)	0.541
21–30	2 (13.3)	11 (18.0)		0 (0.0)	11 (18.0)	
31–40	3 (20.0)	5 (8.2)		2 (25.0)	10 (16.4)	
41–50	5 (33.3)	13 (21.3)		4 (50.0)	14 (23.0)	
51–60	1 (6.7)	8 (13.1)		1 (12.5)	12 (19.7)	
61–70	2 (13.3)	11 (18.0)		0 (0.0)	6 (9.8)	
>70	1 (6.7)	7 (11.5)		1 (12.5)	6 (9.8)	
***Sex***			0.945			0.592
Male	6 (40.0)	25 (41.0)		5 (62.5)	32 (52.5)	
Female	9 (60.0)	36 (59.0)		3 (37.5)	29 (47.5)	
***MS***			0.138			0.150
Single	3 (20.0)	14 (23.0)		1 (12.5)	19 (31.1)	
Married	8 (53.3)	39 (63.9)		6 (75.0)	33 (54.1)	
Divorced	3 (20.0)	2 (3.3)		0 (0.0)	8 (13.1)	
Widowed	1 (6.7)	6 (9.8)		1 (25.0)	1 (1.6)	
***Occupation***			0.796			0.933
Unemployed	2 (13.3)	12 (19.7)		1 (12.5)	8 (13.1)	
Informal	10 (66.7)	33 (54.1)		6 (75.0)	42 (68.9)	
Formal	2 (13.3)	8 (13.1)		1 (12.5)	8 (13.1)	
Student	1 (6.7)	8 (13.1)		0 (0.0)	3 (4.9)	
***BP***			0.321			0.869
Optimal	6 (40.0)	23 (37.7)		2 (25.0)	12 (19.7)	
Normal	2 (13.3)	13 (21.3)		3 (37.5)	19 (31.1)	
Prehypertension	1 (6.7)	13 (21.3)		2 (25.0)	14 (23.0)	
Hypertension	5 (33.3)	8 (13.1)		1 (12.5)	8 (13.1)	
ISH	1 (6.7)	4 (6.6)		0 (0.0)	8 (13.1)	
***Smoking***						0.085
No	13 (86.7)	60 (98.4)	0.037	7 (87.5)	60 (98.4)	
Yes	2 (13.3)	1 (1.6)		1 (12.5)	1 (1.6)	
***Alcohol intake***			0.358			0.812
No	11 (73.3)	51 (83.6)		6 (75.0)	48 (78.7)	
Yes	4 (26.7)	10 (16.4)		2 (25.0)	13 (21.3)	
***SBP (mmHg)***	130.07±31.14	125.38±22.35	0.504	118.37±16.94	128.56±15.29	0.085

Table 5 summarises the associated of the diagnoses of the medical and surgical wards at hospitalisation. There was a significant association between the type of AKI, type of ward, socio-demographic and clinical characteristics of the studied participants.

**Table 5:** Association of diagnosis at hospitalisation with AKI among medical and surgical patients

	Medical (N=76)				Surgical (N=69)		
					
	Yes	No	P-value		Yes	No	P-value
***Diagnosis***			0.030	***Diagnosis***			0.758
Liver Disease	5 (33.3)	8 (13.1)		Hypertension	0 (0.0)	1 (1.6)	
Diabetes	0 (0.0)	7 (11.5)		Respiratory	0 (0.0)	2 (3.3)	
Hypertension	6 (40.0)	5 (8.2)		GI disorder	3 (37.5)	13 (21.3)	
Cardiac							
disease	0 (0.0)	12 (19.7)		Dementia	0 (0.0)	1 (1.6)	
SCD	1 (6.7)	7 (11.5)		Tumour	0 (0.0)	9 (14.8)	
Lung disease	1 (6.7)	7 (11.5)		Goitre	0 (0.0)	3 (4.9)	
Sepsis	0 (0.0)	2 (3.3)		Injury	1 (12.5)	14 (23.0)	
Alcoholism	0 (0.0)	3 (4.9)		Others	4 (50.0)	18 (29.5)	
Anaemia	0 (0.0)	3 (4.9)					
Cranial disorder	0 (0.0)	1 (1.6)					
Others	2 (13.3)	6 (9.8)					

SCD: Sickle Cell Disease

## Discussion

This study assessed the incidence of AKI among medical and surgical patients admitted to a tertiary hospital in Ghana. AKI was found in 15.9% of the participants, with a higher incidence among the medical patients than the surgical patients.

The overall incidence of AKI among the participants was 15.9%. This is higher than the 1.7% incidence reported by Hoste et al. 5 among hospitalized patients in West Africa. Effa et al. 20 at the University of Calabar Teaching Hospital in Nigeria also reported a 3.6% incidence among hospitalized patients. However, Wang et al. 21 at the University of Alabama Birmingham Hospital, USA found the incidence of AKI among hospitalized patients to be 22.7%. The differences in incidence rates could be due to the differences in population and AKI definition criteria used.

Among the participants who developed AKI in this study, stage 1 AKI was found in 56.5% (13/23), with stage 2 and stage 3 AKI present in 26.1% (6/23) and 17.4% (4/23) respectively. Wang et al. 21 observed 15.8% with stage 1, 2.7% with stage 2, and 4.2% with stage 3 when the AKIN criteria was used. On the other hand, et al. 20 found none of the AKI participants having stage 1 with 40.5% and 59.5% having stage 2 and stage 3 AKI respectively. Osman et al. 13 also found that 24.2% of AKI patients were having AKI stage 1, 27.9% AKI stage 2 and 47.9% AKI stage 3 among Sudanese adults admitted to a tertiary level hospital. The use of all hospitalized patients by Effa et al. 20 and Osman et al. 22 as opposed to the use of only medical and surgical patients in this study might have contributed to the finding of majority of participants with AKI having stage 3 in their studies.

About 20% of our participants in the medical ward developed AKI. Effa et al. 20 found 61.9% of their patients with AKI admitted to the medical wards while Osman et al. 22 found 83.8% admitted to the medical wards. This suggests a higher predisposition of patients admitted at medical wards to AKI.

Acute kidney injury (AKI) is a major cause of morbidity and mortality among patients undergoing major surgical interventions worldwide and contributes to prolonged hospital stays and increased cost of treatments. This has been associated with hemodynamic changes and drugs used pre- and post-operative 23. Among adult patients undergoing major surgery in a tertiary hospital in Nigeria, AKI occurred in 18.7% and 17.4% within 24 hours and on day 7 post-op, respectively, with a cumulative AKI incidence at 1-week post-op being 22.5%^8^. This is higher than the 12% observed among surgical patients in this study. However, Mizota et al. 24 in Japan observed a 6.3% incidence among patients undergoing major abdominal surgery. This study and others conducted elsewhere heightens the need for surgeons to conduct a meticulous preoperative risk assessment for AKI, in order to prevent and manage early cases.

The higher incidence of AKI among patients admitted at the medical ward than the surgical ward is consistent with the study by Osman et al. 22 in which more of the patients that developed AKI were admitted to the medical ward than the surgical ward. Also, the study by Effa et al. 20 in Nigeria also reported a higher incidence of AKI among patients admitted to the medical wards than surgical wards. This could be due to the fact that most of the patients in the medical ward had conditions like hypertension and liver disease which could predispose them to AKI. Among the participants admitted to the medical ward, 60.0% (9/15), 26.7% (4/15) and 13.3% (2/15) had stages 1, 2 and 3 AKI respectively, while among the surgical patients, 50.0% (4/8), 25.0% (2/8) and 25.0% (2/8) developed stages 1, 2 and 3 AKI respectively. A study in India by Singh et al.^25^ identified malignancies as the major cause of AKI in patien ts admitted to surgical wards. This is in contrast to the findings of the present study which recorded GI to disorders as the major cause of AKI in the surgical wards. Hypertension has been associated with the development of AKI, and contributes to mortality in the affected patients. Hypertension has been linked to the use of anti-hypertensive agents such as angiotensin converting enzyme inhibitors (ACEI) and presence of haemodynamic changes 12,26. The foregoing was corroborated by the findings of our study in which about one-third of the participants with AKI on the Medical wards were diagnosed of hypertension. Considering the fact that we ensured participants with hypertension or on anti-hypertensives with a previous history of CKD were excluded, our observation further highlights the role of hypertension in the development of acute kidney injury among medical in-patients.

AKI commonly occurs in patients with chronic liver disease and remains a major clinical problem with devastating complications^27^. Almost 20% of people hospitalized with cirrhosis developed AKI, with mortality rates of 50% to 90% 28. This is buttressed by the finding of liver disease as the second leading cause of AKI among the medical patients in this study. Osman et al.^22^ also found chronic liver disease as a major risk factor for AKI in Sudan.

The findings from this study indicates that AKI is high among surgical and medical patients, with hypertension and liver disease as major contributing comorbidities. We therefore recommend that kidney function of persons with hypertension and liver diseases should be assessed regularly by attending clinicians in order to detect any sudden decline in function early. This will help initiate early treatment before progression to chronic kidney disease or end-stage kidney disease requiring renal replacement therapy.

This study, though the first to assess AKI among in-patients at the Cape Coast Teaching Hospital, is limited by study design (prospective cross-sectional) small sample size, single measurement of creatinine and non-standardization of serum creatinine to isotope dilution mass spectrometry (IDMS).

## Conclusion

The incidence of AKI is high among patients admitted to the medical and surgical wards of the Cape Coast Teaching Hospital. Hypertension and liver disease are major comorbidities contributing to the development of AKI. The kidney function of persons with hypertension and liver diseases therefore needs to be monitored regularly in order to detect AKI early and management therapies initiated.

Considerable proportion of surgical patients also suffer AKI. Hence, surgeons should assess their patients for risk of AKI preoperatively and promptly diagnose and manage the condition.

## Figures and Tables

**Table 3:** Socio-demographic, clinical and biochemical characteristics of study participants with AKI, across the wards of admission

	AKI		P-value
	
Variable	Medical (N=15)	Surgical (N=8)	
***Age (years)***	44.7±16.8	46.7±11.3	0.757
***Age group (years)***			0.743
≤20	1 (6.7)	0 (0.0)	
21–30	2 (13.3)	0 (0.0)	
31–40	3 (20.0)	2 (25.0)	
41–50	5 (33.3)	4 (50.0)	
51–60	1 (6.7)	1 (12.5)	
61–70	2 (13.3)	0 (0.0)	
>70	1 (6.7)	1 (12.5)	
***Sex***			
Male	6 (40.0)	5 (62.5)	0.304
Female	9 (60.0)	3 (37.5)	
***MS***			
Single	3 (20.0)	1 (12.5)	
Married	8 (53.3)	6 (75.0)	0.498
Divorced	3 (20.0)	0 (0.0)	
Widowed	1 (6.7)	1 (25.0)	
***Occupation***			0.898
Unemployed	2 (13.3)	1 (12.5)	
Informal	10 (66.7)	6 (75.0)	
Formal	2 (13.3)	1 (12.5)	
Student	1 (6.7)	0 (0.0)	
***BP***			0.344
Optimal	6 (40.0)	2 (25.0)	
Normal	2 (13.3)	3 (37.5)	
Prehypertension	1 (6.7)	2 (25.0)	
Hypertension	5 (33.3)	1 (12.5)	
ISH	1 (6.7)	0 (0.0)	
***Smoking***			0.955
No	13 (86.7)	7 (87.5)	
Yes	2 (13.3)	1 (12.5)	
***Alcohol intake***			0.931
No	11 (73.3)	6 (75.0)	
Yes	4 (26.7)	2 (25.0)	
***SBP (mmHg)***	130.07±31.14	118.37±16.94	0.338
***DBP (mmHg)***	80.20±16.79	75.37±11.45	0.477
